# Therapeutic Plasma Exchange in Myasthenia Gravis: A Systematic Literature Review and Meta-Analysis of Comparative Evidence

**DOI:** 10.3389/fneur.2021.662856

**Published:** 2021-08-31

**Authors:** Tina S. Ipe, Adeola R. Davis, Jay S. Raval

**Affiliations:** ^1^Department of Pathology and Laboratory Medicine, University of Arkansas for Medical Sciences, Little Rock, AR, United States; ^2^Terumo Blood and Cell Technologies, Lakewood, CO, United States; ^3^Department of Pathology, University of New Mexico, Albuquerque, NM, United States

**Keywords:** plasmapheresis, myasthenia (myasthenia gravis-MG), autoimmune disorders, neurological diseases, therapies and management

## Abstract

**Background:** Patients with Myasthenia Gravis (MG) can be treated acutely with therapeutic plasma exchange (TPE) or intravenous immune globulin (IVIG). To date, there is no definitive understanding of which of the two treatments is more effective and safer. The purpose of this study was to systematically review the literature on the comparative efficacy and safety of TPE to other available treatments for MG.

**Methods:** A systematic literature search for studies published between 1997 and 2017 was performed per Preferred Reporting Items for Systematic Reviews and Meta-Analyses (PRISMA) guidelines using two database sources, MEDLINE (through the PubMed database) and Cochrane Library.

**Results:** The search strategy resulted in 535 articles whose abstracts were reviewed. Among these, 165 full texts articles were reviewed for eligibility and 101 articles were excluded. Of the 165 articles, 64 articles were included for a systematic literature and 11 articles for a meta-analysis.

**Conclusions:** This systematic literature review and meta-analysis of treatment options showed that there was a higher response rate with TPE than IVIG in acute MG patients and patients undergoing thymectomy. There was no difference in mortality between the two treatment options. Our findings highlight the need for additional randomized clinical trials in these patients with MG.

## Introduction

Myasthenia gravis (MG) is an autoimmune, neuromuscular transmission disease characterized by a fluctuating weakness of skeletal muscle. It is estimated to affect about 60,000–80,000 people in the United States and about 700,000 people worldwide (1, 2). The disease is clinically classified into two main categories: restrictive ocular MG commonly affecting ocular muscles and generalized MG affecting multiple muscle sets including, but not limited to, ocular muscles. Generalized MG, a focus of this review, accounts for about 80% of all MG (3). MG is commonly caused by antibodies that target the receptors at the neuromuscular junction and is often defined based on those targeted receptors, such as acetylcholine receptor positive (AChR+) MG (~85% of MG), muscle-specific tyrosine-kinase receptor positive (MuSK+) MG (~5% of MG) and low-density lipoprotein receptor-related protein 4 positive (LRP4+) MG (~3% of MG) (4–7). In MG with no detectable levels of any antibodies (~7% of MG), referred to as seronegative MG, the source of immune attack is unclear but the clinical manifestation, treatment options, and treatment response are similar to those of seropositive MG patients.

The hallmark of the disorder is a fluctuating degree and variable combination of weakness in ocular, bulbar, limb, and respiratory muscles. Weakness is the result of an antibody-mediated, T-cell dependent immunological attack directed at proteins in the postsynaptic membrane of the neuromuscular junction (acetylcholine receptors and/or receptor-associated proteins). Thus, MG patients typically exhibit symptoms such as drooping eyelids (ptosis), blurred or double vision (diplopia), change in facial expression, difficulty swallowing, impaired speech, shortness of breath, and progressive muscle weakness of the limbs (7). Because disease progression is gradual and symptoms fluctuate, diagnosis is often delayed in many patients by months or even years. Disease severity rises with the number of muscles affected from moderate affecting only a few muscles to severe affecting multiple muscle groups. MG is typically a chronic condition, yet about 15–20% of patients experience severe acute symptoms within 2 years of diagnosis (3). Disease burden in acute patients is significant as these patients can experience impaired breathing; in some cases, these patients require emergency ventilation due to weakening of breathing muscles leading to a condition called myasthenic crisis or MG crisis.

The diagnosis of MG is based on results of one or more physical and neurological examinations as well as tests such as edrophonium test, blood test, electrodiagnostics, diagnostic imaging, and pulmonary function testing (7). Treatment options vary depending on the patient's state at the time of presentation. Treatment in chronic MG patients is directed at symptom management and typically includes anticholinesterase inhibitors as well as immunosuppressive agents, such as corticosteroids, azathioprine and cyclosporine (4, 7). For the ~15% of MG patients that have a tumor in their thymus they often undergo thymectomy to reduce the risk of myasthenic crisis and improve disease prognosis (8). Acute or severe patients generally receive short term disease stabilizing therapies such as therapeutic plasma exchange (TPE) and intravenous immunoglobulin (IVIG) (9, 10). Management of acute patients is critically important because failure to intervene with a proper treatment option can lead to respiratory failure, paralysis, and potentially death. TPE stabilizes acute patients by removing the destructive antibodies; it does this by replacing plasma containing harmful antibodies with a disease-free replacement fluid (either albumin or plasma) (11, 12). The mechanism of action of IVIG has yet to be fully elucidated. However, it has been shown that IVIG, when injected, directly binds to the disease-causing antibodies and neutralizes them for relief from immune attack (13).

TPE, the focus of this review, is a type of apheresis therapy in which plasma that contains disease-causing antibodies is separated and removed from the patient's bloodstream. The TPE procedure can take place through central or peripheral venous access points. There are several TPE systems available from different manufacturers. In the treatment of acute MG patients, including but not limited to myasthenic crisis, TPE, and IVIG are often used interchangeably. Despite several literature reviews (14–25) on TPE, IVIG, or both, there is not a clear agreement as to which of the two acute therapy options is more effective or safer; results can vary across studies and patient types. The decision to use TPE vs. IVIG may also depend on factors such as access and convenience.

The purpose of this literature review and meta-analysis is to assess the comparative efficacy and safety of TPE against available treatment modalities and/or untreated patients.

## Materials and Methods

### Literature Search and Review

The systematic literature review was conducted per Preferred Reporting Items for Systematic Reviews and Meta-Analyses (PRISMA) guidelines with two independent database sources, MEDLINE (through the PubMed database) and Cochrane Library (26). A systematic search of both database sources was conducted for studies published between 1997 and 2017 using predefined search terminology focusing on TPE and MG. Table 1 presents search terms and combinations used to search both databases. In the report, therapeutic plasma exchange was used unless a reference did not specify the specific type of plasmapheresis. “Plasmapheresis” is a slightly broader term, which includes TPE, and was used within the report only when a particular type of plasmapheresis was not specified in a cited reference. Only studies published in English addressing the efficacy and/or safety of TPE in MG were included. Efficacy endpoints to be included were pre-defined prior to the full text review (Table 2). Safety endpoints, on the other hand, were not pre-defined; all available data were captured except for those reported as “any adverse event (AE)” or “any complication” rates due to the variable or omitted definitions for these terms in many publications. Original clinical research articles and consensus guidelines were included for use in the report. Systematic reviews & meta-analyses were included for context and as potential sources of additional references. All other publication types were excluded: non-systematic reviews, unofficial guidelines, preclinical research, and journal article comments. Abstracts of all studies identified through the search were scored by two independent reviewers to assess eligibility for inclusion.

**Table 1:** Search terminology.

**MG terms**		**TPE terms**
“myasthenia gravis” OR	**AND**	“TPE” OR
“congenital myasthenia” OR		“therapeutic plasma exchange” OR
“acquired myasthenia” OR		“plasmapheresis” OR
“myasthenia” OR		“plasma exchange” OR
“myasthenic” OR		“PLEX” OR
“myasthenic crisis” OR		“Optia” OR
“myasthenic syndromes”		“CORE” OR “Spectra”

**Table 2:** Efficacy endpoints of interest.

**Category**	**Endpoint**
Disease severity score	Quantitative Myasthenia Gravis Scale (QMGS)
	Myasthenia Gravis Composite (MGC) Scale
	Myasthenia Gravis-Activities of Daily Living (MG-ADL)
	Myasthenia Gravis Foundation of America (MGFA) Score
	MGFA- Post Intervention Status (MGFA-PIS)
	Myasthenia Gravis Disease Scale (MGDS)
	Myasthenic Muscle Score (MMS)
	Myasthenia Gravis Severity Scale (MSS)
	Osserman classification
	Other/unspecified scale
Quality of Life (QOL)	Short Form 36 (SF-36)
	QOL-60
	QOL-15
	Other QOL scales
Speed of recovery	Ventilation time
	Hospitalization time
Serological factors	AChR antibody titer
	MuSK antibody titer
Other	Re-hospitalization rate
	Recurrent crisis rate
	Prednisone dose
	Electromyogram (EMG)
	Repetitive nerve stimulation

### Study Selection Process

**Figure 1:**
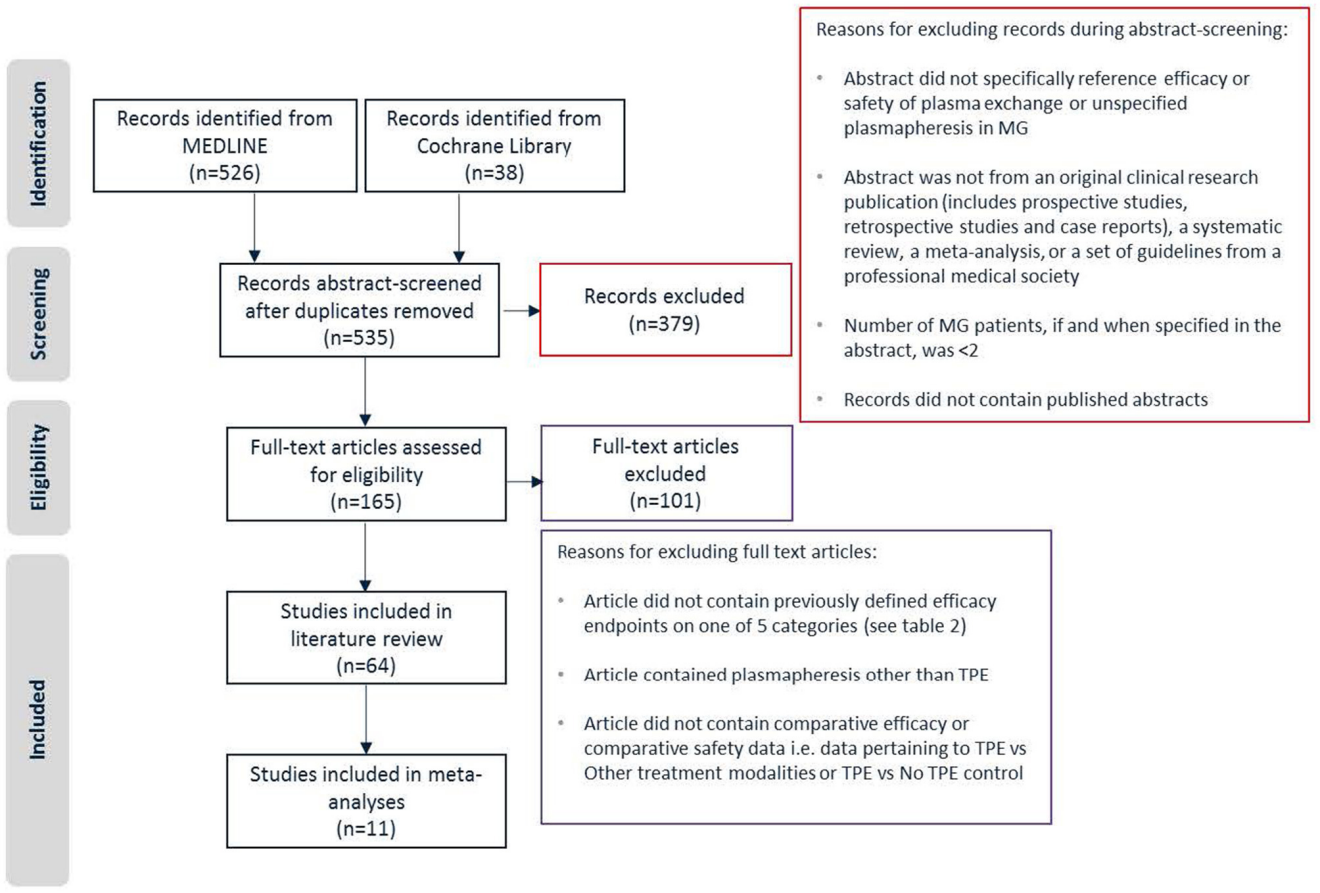
Study selection.

A total of 526 studies from MEDLINE and 38 studies from the Cochrane Library were identified based on search terminology. Following the removal of duplicates, 535 papers were selected for abstract screening. Abstract screening led to a total of 165^1^ articles as eligible candidates for full text review. Bibliographies were checked for additional references, including pre-1997 publications. In cases where an article was not available for download, inquiries were sent to the publisher and corresponding author. Despite these efforts, 3 articles could not be obtained for full text review (27–29). All eligible papers were reviewed and a total of 64 papers met the final criteria and were included in the literature review. Of these 64 papers, 13 cited one or more specific TPE systems, including 6 that cited the COBE Spectra Apheresis System and 1 that cited the Spectra Optia Apheresis System (Terumo BCT, Inc., Lakewood, CO, USA; formerly Caridian BCT). There were numerous non-comparative studies of TPE in MG that are not included in this review. Figure 1 presents the study selection process applied during abstract screening and full text review.

### Data Extraction

During data extraction, reviewers noted and discussed potential sources of biases including imbalances in baseline characteristics, non-randomized nature of studies, and unmasked outcomes. In some cases where data was presented graphically, but not in explicit numerical form, WebPlotDigitizer was used to extract data points from figure images. Where available, statistical summaries including *p*-values from studies are reported. Where original publications did not provide a test of statistical significance, *p*-values were calculated using Fisher's exact test for categorical data and student's *t*-test for continuous data, if appropriate. Continuous variables are described as mean ± standard deviation unless otherwise noted.

### Meta-Analysis

All efficacy and safety endpoints captured by 36 comparative safety and comparative efficacy papers were qualified as candidates for meta-analysis. The decision to run a meta-analysis on a specific endpoint was based on two criteria: a) there must be at least 3 papers containing data on that endpoint and b) available data on that endpoint across studies must contain a matching comparator treatment and a comparable study background including treatment context, age group, and outcome measures. Based on these criteria, two endpoints qualified for the meta-analysis. Both compare TPE and IVIG in acute MG patients, with one analysis focused on response rate and the other mortality rate.

Meta-analysis was performed using a random-effects model to account for the fact that the treatment effect may vary due to a variation in patient populations across studies. The outcome of the meta-analysis was reported as risk differences between TPE and IVIG along with 95% confidence intervals (95% CI). The model was fit using the DerSimonian and Laird method with a continuity correction of 0.5 in studies with 0 cell frequencies (30). Bias in meta-analyses was assessed using Egger's method (31).

## Results

### Professional Guidelines and Guidance Statements on the Use of TPE in MG

Three sets of prominent US and EU professional guidelines & guidance statements recommend the use of TPE in one or more MG clinical situations (2, 4, 32–36). The most recent guidance from the MG Foundation of America (MGFA) Task Force and guidelines from the European Federation of Neurological Societies (EFNS) and American Society for Apheresis (ASFA) all recommend the use of TPE in cases of severe acute MG, including myasthenic crisis, and in preparation for thymectomy. The ASFA guidelines go further to include patients with moderate disease severity. The MGFA Task Force recommendations expand the set of appropriate clinical situations to include maintenance treatment in juvenile, refractory, or immunosuppressant-contraindicated patients, short term treatment during pregnancy, or any other time that a rapid response is required.

In contrast to the references above that recommend the use of TPE in MG, 2011 guidelines from the American Academy of Neurology (AAN) conclude that there is not enough evidence to support or refute the use of TPE in MG citing a lack of randomized, controlled clinical trials with masked outcomes (37)^2^. However, the AAN guidelines acknowledge that TPE is being used at many medical centers for the treatment of myasthenic crisis and MG pre-thymectomy.

The MGFA Task Force, ASFA, and EFNS provide further commentary on the tradeoffs between TPE and IVIG. All three guidelines describe TPE and IVIG as equally effective, but tentatively suggest potential advantages for each. The MGFA Task Force guidance and EFNS guidelines state that TPE may work faster in general and may be more effective in MuSK+ patients than IVIG (2, 33). The MGFA Task Force guidance and ASFA guidelines describe a more favorable safety profile for IVIG compared to TPE (2, 4). No strong recommendation for one over the other is given, though EFNS guidelines state that IVIG may be preferred due to fewer and less severe side effects, while the MGFA Task Force says that expert consensus suggests that TPE is more effective.

Recommendations from on optimizing TPE outcomes include the use of peripheral rather than central venous access (2), early rather than delayed initiation of TPE during crisis (33), and delay of corticosteroid treatment during crisis until initial improvement is achieved via TPE (2). The guidelines are careful to note that although the clinical effect of TPE is rapid (1–7 days), durability is limited & variable (2–12 weeks).

Older (38) or regional (39) guidelines on the use and limitations of TPE in MG have also been published. Consensus statements recommending TPE as a valuable treatment for both acute MG crisis and pre-thymectomy date back at least as far as 1986 (38). A summary of German Society for Neurology guidelines notes that TPE, IVIG, and immunoadsorption (IA) are equivalently recommended for the treatment of myasthenic crisis (40).

### TPE Efficacy

#### Efficacy of TPE in the Treatment of Acute MG

Acute MG patients require immediate medical attention to prevent worsening of symptoms and possibly death. There is a large body of evidence associating TPE with improved disease severity and recovery from crisis in acute MG patients (5, 41–46). In comparison to IVIG, meta-analysis results indicate a higher overall response rate in patients treated with TPE. Shorter ventilation times have been observed with TPE, while shorter overall hospitalization times have been reported with IVIG. However, for most endpoints, including QOL scores, response time, electrophysiological metrics, and antibody titers, no significant evidence exists for a difference between the two treatments. It is important to note that studies comparing TPE to “no treatment” are not found in the literature likely due to the seriousness of acute MG and the timeframe in which the treatment was developed.

##### TPE vs. IVIG

Numerous studies have compared the effectiveness of TPE and IVIG in the treatment of acute MG. The majority of these, comprising 2 prospective, randomized trials (5, 41) and 4 retrospective analyses (42–44, 47) included a measure of the impact of TPE and IVIG on overall disease burden. All response rates to TPE treatment were ≥50% of patients and all changes from baseline on established MG disease severity scales were statistically significant. In each of the 6 studies, reported response rates and/or mean response magnitude was greater among patients treated with TPE than among those treated with IVIG^3^. However, the difference reached statistical significance in only one of the studies (44).

To further explore the difference in efficacy across studies, a meta-analysis of response rates for TPE vs. IVIG in acute MG was performed (Table 3). Data from 4 studies were determined to be sufficiently comparable for inclusion in the analysis (see Table 4 for study design summaries). Studies with response rates that were not based on a specific disease severity or outcome scale were excluded (42, 47). The summary statistic, response risk difference (TPE % responders minus IVIG % responders), ranged from +7% to +32% for the 4 eligible studies; a positive risk difference indicates that more patients responded to TPE vs. IVIG. The pooled estimate based on a random effects model was a +19% response risk difference in favor of TPE vs. IVIG in acute MG. This result was statistically significant (*p* = 0.002). Egger's test did not indicate bias (*p* = 0.6729), but the small number of studies used limits the power of this assessment (31).

**Table 3:** Meta-analysis results comparing response risk differences between TPE and IVIG in acute MG patients.

**Reference**	**No. of patients**	**Relative weight**	**Response Risk difference: TPE % responders–IVIG % responders**	
Ramos-Fransi et al. (43)	17	6%	14% (95% Cl: −35%, 63%)	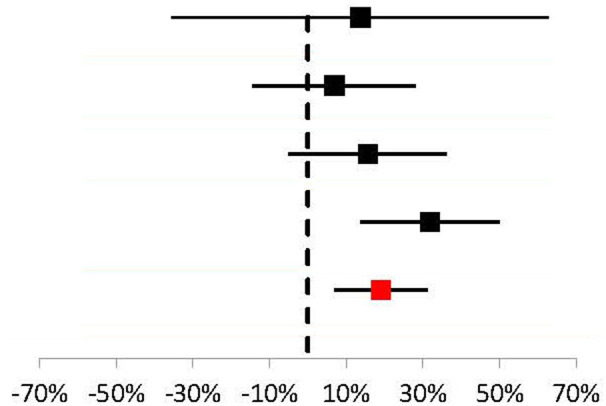
Barth et al. (5)	84	28%	7% (95% Cl: −14%, 28%)	
Gajdos et al. (41)	87	29%	16% (95% Cl: −5%, 36%)	
Guptill et al. (44)	104	37%	32% (95% Cl: 14%, 50%)	
Pooled	292		19% (95% Cl: 7%, 31%)	

**Table 4:** TPE vs. IVIG response rates and sizes in acute MG data.

**Reference**	**Ramos-Fransi et al. (43)**	**Guptill et al. (44)**	**Barth et al. (5)**	**Murthy et al. (47)**	**Qureshi et al. (42)**	**Gajdos et al. (41)**
Treatment context	Retrospective	Retrospective	Prospective	Retrospective	Retrospective	Prospective
Comparison	Acute	Acute	Acute	Acute	Acute	Acute
Study type	Severe	Mild to severe	Moderate	Severe	Severe	Moderate to severe
Severity	AChR+ (90%)	MuSK+ (100%)	AChR+ (75%)	Not specified	Not specified	AChR+ (70%)
Common serotype	TPE/IVIG	TPE/IVIG	TPE/IVIG	TPE/IVIG	TPE/IVIG	TPE/IVIG
# Patients	6/11	73/31	43/41	15/8	28/26	41/46
Timing (response rate)	30 days	Not specified	14 days	At discharge	28 days	15 days
Response rate	MGFA-PIS: 50/36%	MGFA-PIS: 93/61%^∧^	ΔQMGS ≥ 3.5: 58/51% MGFA-PIS: 65/ 69%	Clinician definition of complete resolution: 93/88%	Clinician definition of complete resolution: 71/62%	ΔMMS ≥ 20: 63/48%
Timing (response size)	-	-	14 days	-	7 days	15 days
Response size	-	-	ΔQMGS:4.7 ± 4.9/3.2 ± 4.1^*^	-	ΔMSS: 4.2/2.8	ΔMMS: 16.6 ± 5.0/15.6 ± 4.7 (95% Cl)^*^

*No significant difference observed between treatments (p ≥ 0.05) unless noted*.

∧ *p < 0.05 as reported by original authors or calculated using Fisher exact test*.

* *Combined data from two separate IVIG groups: 3-day IVIG treatment 18.9 (95% Cl: 13.1, 24.7) and 5-day IVIG treatment 12.4 (95% Cl: 5.0, 19.8)*.

When comparing TPE to IVIG for other key endpoints, published evidence shows limited differences between the two treatment modalities. However, evidence suggests that compared to IVIG, patients treated with TPE experience shorter ventilation times but longer hospitalizations.

In general, studies comparing TPE and IVIG have not found significant differences for most endpoints. For example, improvements in quality of life scores (48), response times (41, 42), electrophysiological improvement (5), and decrease in AChR antibody titers (41) are all key endpoints for which no significant differences between TPE and IVIG have been reported in acute MG. However, there is indirect evidence that responses to TPE may be faster (42)^4^, but less durable (5), than responses to IVIG based on response time and electrophysiological data, respectively. Direct comparison of response times also showed a noticeably shorter median response time for TPE vs. IVIG, though the difference did not reach statistical significance (42).

In contrast, speed of recovery, as measured by ventilation and hospitalization times, is an area in which significant differences between TPE and IVIG have been observed. Specifically, a higher rate of early extubation and superior ventilation status at 2 weeks was observed for TPE vs. IVIG in one study (42). Conversely, significantly longer hospitalization times were observed among MG crisis patients treated with TPE vs. IVIG (1). For both of these endpoints, there are studies providing indirect (49) or directional (42, 50) support for these conclusion.

##### TPE vs. Other Treatment Modalities

In addition to IVIG, the efficacy of TPE has been compared to other treatment modalities in acute MG including immunoadsorption and as an addition to other treatment backgrounds. In 2 studies comparing TPE to IA in acute MG, no significant differences between the two treatments were observed (51, 52). However, the combination of TPE + immunoadsorption was associated with significantly shorter hospitalization times than TPE alone (51).

Studies of other treatment backgrounds with or without TPE have either found better outcomes with TPE (49) or did not observe significant differences (53, 54) among patients receiving treatment for acute MG.

### Efficacy of Pre-thymectomy TPE in MG

Studies have found pre-operative TPE to be associated with better thymectomy outcomes. However, there is not strong evidence to support TPE as a superior treatment to IVIG in the pre-thymectomy setting.

#### TPE vs. No Immunomodulatory Treatment

The majority of published evidence has demonstrated that outcomes in patients undergoing thymectomy are superior for those who receive TPE prior to surgery compared to those who do not receive immunomodulatory therapy (Table 5). Retrospective studies comparing TPE to no pre-thymectomy treatment have found that TPE significantly increases speed of post-operative recovery (55, 57), improves long-term response rate and magnitude (10, 55) and decreases incidence of crisis during follow up (10, 55). The single exception to this trend was a retrospective study in which a lower immediate extubation rate and longer hospitalization time were reported for TPE, though in neither case were the differences statistically significant (56).

**Table 5:** Pre-thymectomy TPE comparative efficacy data.

**References**	**Nagayasu et al. (10)**	**Sarkar et al. (55)**	**Saeteng et al. (56)**	**d'Empaire et al. (57)**	**Jensen and Bril (58)**	**Alipour-Faz et al. (9)**
Treatment Context	Pen-operative	Peri-operative	Peri-operative	Peri-operative	Peri-operative	Peri-operative
Comparison	TPE/No TPE	TPE/No TPE	TPE/No TPE	TPE/No TPE	TPE/IVIG	TPE/IVIG
Study Type	Retrospective	Retrospective	Retrospective	Retrospective	Retrospective	Prospective
Severity	Mild to moderate	Moderate to severe	Mild to moderate	Not specified	Mild to moderate	Not specified
Common serotype	AChR+ (75%)	AChR+ (100%)	Not specified	Not specified^***^	Not specified	AChR+ (100%)
# Patients	19/32	10/9	33/53	11/26	9/9	12/12
Response time point	5–7 yrs	1 yrs	-	-	1st post-op visit	-
Response rate	MGFA-PIS: 100/81.3% ^∧^	Osserman = 0: 80/44%	-	-	Δ Osserman −1 or better: 78/56%	-
		Δ Osserman:			Δ Osserman:	
Response magnitude	-	−2.7 ± 0.5 /	-	-	−1.00 ± 0.71/	-
		−1.7 ± 0.7 ^∧^			−0.78 ± 0.83	
Ventilation time (days unless stated otherwise)	-	1.3 ± 0.4/4.7 ± 3.2 ^∧^	Immediate extubation rate: 88/94%	1.02 ± 0.4/3.43 ± 0.6 ^∧^	-	0.54 (0.08–9)/0 (0.00^*^-0.92) ^∧^^**^
Hospitalization time		4.7 ± 1.2/	6.1 ± 4.2/		3.3 ± 1.2/	20.3 ± 8.4/
(days)		9.0 ± 3.9 ^∧^	5.2 ± 2.3		2.2 ± 1.9	21.1 ± 5.2
Crisis incidence timing	1 yr	1 yr	-	-	-	hosp.
Crisis incidence rate	5.3/28.1% ^∧^	0/33%	-	-	-	17/0%

*Abbreviations: hosp., hospitalization*.

*No significant difference observed between treatments (p ≥ 0.05) unless noted*.

∧ *p < 0.05 as reported by original authors or calculated using Fisher exact test or student's t-test, if appropriate*.

* *Lower value was updated from 0.08 (as reported in the paper) to 0.00 to include the reported median value (0) within the range*.

** *median (range)*.

*** *Data from abstract, full text was not available despite requests to corresponding author and publisher*.

#### TPE vs. IVIG

When compared to IVIG in the pre-thymectomy context, there is no available evidence to suggest that TPE leads to superior outcomes. Across one prospective and one retrospective comparison, no significant differences in response rate or magnitude, hospitalization time, or incidence of crisis were observed between TPE and IVIG (9, 58). However, in the prospective study, patients receiving TPE were intubated for a significantly longer time than those receiving IVIG (9).

#### Efficacy of TPE in Chronic MG

##### TPE vs. IVIG

While TPE is routinely used in the treatment of acute MG patients and those undergoing thymectomy, comparative data is sparse in the maintenance treatment of chronic, stable patients. However, in the limited literature for chronic MG that is available, there is data showing higher response rates to TPE vs. IVIG among juvenile patients (25) and no evidence of a significant difference between the two treatments in adults (45).

A controlled crossover trial of TPE and IVIG in chronic, stable adult MG patients tracked QMGS and AChR titers for 16 weeks after an initial course of treatment (45). During the TPE phase, a statistically significant improvement from baseline QMGS was observed at 1 week, maintained at 4 weeks, but was no longer significant by 8 weeks (*p* < 0.05). In contrast, a statistically significant improvement from baseline was not reached until 4 weeks for IVIG and, similarly, was no longer significant by 8 weeks. No statistically significant differences between TPE and IVIG were observed at any timepoint as measured by QMGS. TPE, but not IVIG, led to a statistically significant reduction in AChR titers at 1 week (average decline of 79%, *p* < 0.001)^5^. In the longer timeframe, neither group showed any statistically significant reduction in AChR titers (4–16 weeks).

Additionally, retrospective analysis of maintenance therapies in juvenile MG patients found that the response rate to TPE was significantly higher than IVIG (100 vs. 50%; *p* = 0.04) (25).

##### TPE vs. Other Treatment Modalities

In addition to IVIG, the efficacy of TPE has been compared to other treatment modalities in acute MG including pyridostigmine and as an addition to other treatment modalities, such as glucocorticosteroids. In a prospective study of chronic stable moderate patients treated with pyridostigmine or TPE, significantly larger improvements in several pulmonary function metrics was observed with TPE (59). Studies of steroid treatment backgrounds (i.e., prednisolone, prednisone) with or without TPE have not observed significant differences in maintenance therapy (60, 61). One prospective study of long term TPE and prednisone treatment found a faster response when TPE was added to a background of prednisone, although the trend did not continue over a longer period of 24 months (61); a higher rate of exacerbations was noted in the TPE group, but the difference was not significant.

#### Considerations to Maximize TPE Efficacy

Some studies have looked at ways to optimize TPE efficacy (62–66). For example, published evidence shows that treatment schedule and venous access route can impact TPE efficacy in MG.

##### Treatment Schedule

Timing and frequency of TPE in MG may impact speed of recovery. A randomized trial of acute MG patients reported that patients receiving daily TPE spent a median of 17.5 days in the hospital whereas those receiving TPE on alternate days spent a median of 26 days, though this difference was not statistically significant (*p* = 0.054) (65). Similarly, timing of TPE can affect recovery speed as evidenced by a study which showed that MG crisis patients receiving TPE within 2 days of admission had a significantly shorter hospital stay compared to those receiving TPE >2 days after admission (6 days vs. 14 days; *p* < 0.001) (63).

##### Venous Access Route

Peripheral venous access is associated with faster recovery compared to central venous access. A retrospective analysis of MG patients treated with TPE found that patients who received TPE via peripheral venous access spent significantly less time in the hospital compared to those who received TPE via a central venous line (median: 9 days [range 6–10] versus 12 days [range: 8–18], *p* = 0.002; 84 and 94% acute patients, respectively) (62).

#### Specific Population: MuSK+ Patients

There is a small body of evidence demonstrating the efficacy of TPE specifically in MuSK+ MG patients including evidence of higher response rates with TPE vs. IVIG. A retrospective analysis of 110 MuSK+ acute MG patients reported that 93% of patients who received TPE and 61% of patients on IVIG saw clinical improvements based on MGFA-PIS (*p* = 0.0002) (44). A prospective trial in MuSK+ patients compared the efficacy of TPE (*N* = 3) against that of early prednisone (*N* = 6) and thymectomy (*N* = 3) by grading patients on MGFA classification at the onset of myasthenic symptoms, in the maximally deteriorated state, and at the last clinic visit after or during treatment (60). It found that while all patients on TPE and early prednisone improved (*p* = 1.0), no patient improved post-thymectomy. It is important to note, however, that patients in the prednisone and TPE groups had more severe baselines (IIb to V) than the thymectomy group (all IIb) and differences in response rate for TPE vs. thymectomy in MuSK+ patients did not reach statistical significance (*p* = 0.1). Although other studies exist that highlight the benefits of TPE over IVIG in MuSK+ patients, they do not provide clear *comparative* evidence [i.e., one study reports response rates for TPE and IVIG, but the number of patients who received only TPE, only IVIG, or both TPE and IVIG is not specified (67); another publication studied TPE in patients that had failed IVIG rather than a side by side comparison (68)].

#### COBE Spectra and Spectra Optia Apheresis Systems

As noted, several types of TPE systems are available to perform TPE procedures in MG patients. Although preclinical publications exist, data comparing the clinical efficacy of different TPE systems was not found in published literature. Most comparative papers either use more than one in a single study or do not specify which was used.

Among publications included in this review that specified one or more specific TPE systems, the Spectra line of systems, COBE Spectra and Spectra Optia Apheresis Systems were the most commonly cited (5, 51, 62, 64). Comparative studies that exclusively used both COBE Spectra and Spectra Optia Apheresis Systems include a prospective, randomized trial of TPE vs. IVIG in acute MG (5). In the TPE group, mean QMGS scores were significantly improved at day 14 and this improvement was maintained through day 28 (*p* < 0.0001). The majority of TPE patients responded to treatment according to two separate scales^6^, but no significant difference was observed in response rate to TPE vs. IVIG on either scale (*p* = 0.5 to 0.74). Although mean values of QMGS improvement were greater at all time points for TPE compared to IVIG, none of the differences were statistically significant (*p* = 0.07 to 0.13).

A retrospective analysis from an institution solely using the COBE Spectra Apheresis System for TPE found that patients receiving pre-operative TPE using the COBE Spectra Apheresis System saw improvements following thymectomy compared to those who did not receive a pre-operative TPE (64). Another retrospective analysis, which exclusively used the COBE Spectra Apheresis System, found that peripheral venous access is associated with shorter hospitalizations compared to central venous access among acute MG patients treated via TPE (62). Lastly, a retrospective analysis comparing TPE vs. immunoadsorption in acute MG was published by an institution that utilized COBE Spectra Apheresis System as one of its two TPE systems (AS104 from Fresenius Kabi, Bad Homburg, Germany also used). Findings in the TPE group included a statistically significant improvement in QMGS from baseline to time of discharge (*p* < 0.0001) (51). However, there was no statistically significant difference in efficacy between the TPE and immunoadsorption groups.

### TPE Safety

#### Mortality

No significant increase in mortality risk has been reported for TPE compared to other MG treatment modalities, including IVIG. In the pre-thymectomy context, the use of TPE has not been shown to significantly affect mortality compared to untreated patients. In myasthenic crisis, TPE with corticosteroid treatment is associated with significantly lower mortality than treatment with corticosteroids alone. This report provides a complete summary of TPE in MG studies with comparative all-cause mortality data.

##### TPE vs. No TPE

Most published studies that compare TPE to an untreated group are within the peri-operative context. In a retrospective analysis of patients receiving (*N* = 10) or not receiving (*N* = 9) pre-thymectomy TPE, no deaths were reported among either patient group through 1 year of follow-up (55). Another retrospective analysis compared two pre-thymectomy protocols: a universal protocol in which all patients underwent TPE (*N* = 74) and a selective protocol in which only “high risk” patients underwent TPE (*N* = 90). No deaths were reported under either protocol during hospitalization (64). A third retrospective study, comparing patients receiving (*N* = 33) or not receiving (*N* = 53) pre-thymectomy TPE, reported 1 death during hospitalization in each group and no statistically significant difference between the two options (*p* = 1.00) (56).

One very small prospective study reported outcomes for myasthenic crisis patients treated with intravenous methylprednisone (MP) with or without TPE (54). During hospitalization, mortality was significantly higher in patients treated only with MP compared to those who received both MP and TPE (100% mortality, *N* = 3 vs. 0% mortality, *N* = 4; *p* = 0.03). The authors note that although all patients were mechanically ventilated, some patients were not treated in the ICU due to limited resources. Another, older (1970–1995) retrospective analysis reported high mortality rates among myasthenic crisis patients treated with or without TPE in a background of pyridostigmine ± prednisolone, but there was no significant difference between the +TPE and -TPE groups (19 vs. 10%, *p* = 0.42) (53).

##### TPE vs. IVIG

As with efficacy, studies of TPE vs. IVIG represent the greatest volume of comparative TPE mortality data. No statistically significant differences between the two have been reported across treatment contexts. For example, the largest published cohort is a retrospective analysis of the Healthcare Cost and Utilization Project-Nationwide Inpatient Sample (HCUPNIS) administrative database, which reported in-patient mortality rates across all MG diagnoses & treatment contexts (1). Although the unadjusted mortality rate was higher in TPE than in IVIG (2.6 and 0.6%), the adjusted odds ratio of 2.6 was not found to be statistically significant (*p* = 0.21).

To provide greater strength of evidence within a single treatment context, a meta-analysis of TPE vs. IVIG all-cause mortality in acute MG was performed (Table 6). Data from 7 studies were determined to be sufficiently comparable for inclusion in the analysis (See Table 7 for study design and demographic summary). Chronic, maintenance studies (25, 45) and mixed cohorts (1)^7^ were excluded. Mortality risk difference (TPE mortality % minus IVIG mortality %), ranged from−5.8% to +5.1%. The pooled estimate based on a random effects model was a +1.5% mortality risk difference (higher risk in TPE) but was not statistically significant (*p* = 0.264). Egger's test did not indicate bias (*p* = 0.065), but the small number of studies used limits the power of this assessment (31). Thus, even when aggregating data from all published comparisons, there is insufficient evidence to conclude that TPE and IVIG have different all-cause mortality rates in acute MG.

**Table 6:** Results of meta-analysis (TPE vs. IVIG) of mortality risk difference in acute MG.

**References**	**No. of patients**	**Relative weight**	**Response risk difference: TPE % responders—IVIG % responders**	
Barth et al. (5)	81	19%	0.0% (95% Cl: −4.7%, 4.7%)	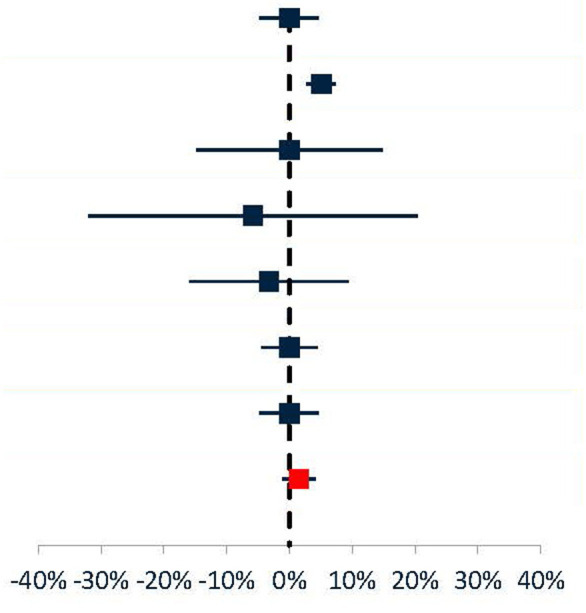
Mandawat et al. (1)	698	34%	5.1% (95% Cl: 2.8%, 7.4%)	
Pittayanon et al. (50)	30	3%	0.0% (95% Cl: −14.9%, 14.9%)	
Murthy et al. (47)	23	1%	−5.8% (95% Cl: −32.0%, 20.3%)	
Qureshi et al. (42)	51	4%	−3.2% (95% Cl: −16.0%, 9.5%)	
Gajdos et al. (41)	87	20%	0.0% (95% Cl: −4.4%, 4.4%)	
Guptill et al. (44)	103	19%	0.0% (95% Cl: −4.7%, 4.7%)	
Pooled	1,073		1.5% (95% Cl: −1.2%, 4.2%)	

**Table 7:** Comparative all-cause mortality data for TPE in MG.

**Reference**	**Treatment context**	**Comparison**	**Timing**	**# Patients**	**Mortality (%)**	***P*** **-value** ^**∧**^
Lal et al. (54)	Acute	TPE/No TPE^**^	hosp.	4/3	0/100%	0.03
Berrouschot et al. (53)	Acute	TPE/No TPE**	3 mo	21/42	19/10%	0.42
Gajdos et al. (41)	Acute	TPE/IVIG	15 days	41/46	0/0%	1.00
Barth et al. (5)	Acute	TPE/IVIG	30 days	41/40	0/0%	1.00
Pittayanon et al. (50)	Acute	TPE/IVIG	hosp.	21/9	0/0%	1.00
Qureshi et al. (42)	Acute	TPE/IVIG	hosp.	24/27	4/7%	1.00
Murthy et al. (47)	Acute	TPE/IVIG	hosp.	15/8	7/13%	1.00
Guptill et al. (44)	Acute	TPE/IVIG	hosp.*	72/31	0/0%	1.00
Köhler et al. (52)	Acute	TPE/IA	180 days	10/9	10/0%	1.00
Schneider-Gold et al. (51)	Acute	TPE/IA	hosp.	19/24	0/0%	1.00
Trikha et al. (65)	Acute	TPE QD/TPE QAD	hosp.	16/17	13/6%	0.59
Rønager et al. (45)	Chronic	TPE/IVIG	16 wk	12/12	0/0%	1.00
Liew et al. (25)	Chronic	TPE/IVIG	1 yr*	17/20	0/0%	1.00
Sarkar et al. (55)	Peri-operative	TPE/No TPE	1 yr	10/9	0/0%	1.00
Saeteng et al. (56)	Peri-operative	TPE/No TPE	hosp.	33/53	3/2%	1.00
Nagayasu et al. (10)	Peri-operative	TPE/No TPE	116 mo*	19/32	0/6%	0.52
El-Bawab et al. (64)	Peri-operative	TPE all pts/TPE selective	hosp.	74/90	0/0%	1.00
Mandawat et al. (1)	Mixed	TPE/IVIG	hosp.	1,269/340	3/1%	0.21^∧∧^
Guptill et al. (62)	Mixed	TPE peripheral/TPE central	treatment	100/34	0/6%	0.07
Mandawat et al. (63)	Mixed	TPE early/TPE delayed	hosp.	870/183	1/7%	<0.0001
Rock et al. (69)	NS	TPE pentastarch/TPE albumin	Not specified	4/3	0/0%	1.00

hosp., during hospitalization; QD, daily; QAD, every other day; lA, immunoadsorption; NS, not specified^*^ Timing: (44) through hospitalization or later, (25) median 1 yr follow-up (range 0–5 yrs), (10) mean 116 mo for all patients (100.2 ± 41.2 mo TPE vs. 125.1 ± 77.5 mo No TPE).

∧ *Calculated by report authors using Fisher's exact test unless otherwise noted*.

∧∧ *P-value for adjusted odds ratio as reported by original study authors*.

** *Background treatment: (54) intravenous methyl prednisone, Berrouschot et al. (53) pyridostigmine or pyridostigmine + prednisolone*.

Mortality data has also been published in the chronic/maintenance setting, though the number of published comparisons of TPE vs. IVIG is far fewer than in acute MG. In one retrospective analysis, 27 juvenile MG patients were treated with TPE or IVIG every other week, with tapering if possible (25). Over a median 1-year follow-up, no deaths were reported in either group. Lastly, in a prospective controlled crossover trial, 12 stable, chronic MG patients were treated with a course of TPE or IVIG (45). Through 16 weeks of follow-up, no deaths were reported in either group.

##### Optimizing TPE

A few studies have also looked at the effects of procedural factors on mortality in MG patients treated with TPE. In a retrospective analysis of the HCUPNIS administrative database, inpatient mortality was reported for patients receiving early TPE (0–2 days from admission) or delayed TPE (>2 days from admission) under any MG treatment context (63). All-cause mortality was significantly higher in patients who received delayed vs. early TPE (6.6% *N* = 183 vs. 1.2% *N* = 870, *p* < 0.0001: adjusted odds ratio 1.86, *p* < 0.0001).

A retrospective study of the impact of access route on TPE complications in MG compared mortality for peripheral vs. central venous access (62). Across a mix of treatment contexts, no deaths were reported in patients receiving TPE via peripheral venous access (*N* = 100). Two deaths occurred among patients receiving TPE via central access (*N* = 34), but this difference did not reach statistical significance (*p* = 0.07).

##### COBE Spectra and Spectra Optia Apheresis Systems

As noted above for efficacy, data comparing the mortality associated with different TPE systems was not found in published literature. However, Table 7 includes all-cause mortality data from several studies captured in this report that used the Spectra systems, COBE Spectra and Spectra Optia Apheresis Systems, exclusively (5, 62, 64) or as one of two cited systems (51, 69). Across all treatment contexts in the studies using COBE Spectra and Spectra Optia Apheresis Systems exclusively, 2 deaths were reported among 284 treated patients. Both deaths occurred in patients described as having MG-related immobility and were suspected to have been caused by central venous catheter complications: urosepsis and pulmonary embolism (62).

#### Other Adverse Events

Studies which resulted in statistically significant differences between TPE, and any comparator treatment were limited to a handful of publications comparing TPE vs. IVIG. While incidence of certain AEs is greater in TPE, there are other AEs more frequently seen in IVIG. Existing evidence points to peripheral venous access and early treatment as the TPE procedural factors most strongly associated with lower AE rates. A compilation of all vascular, cardiac, infection, and other^8^ AE rates from comparative studies, including those for which significant differences were not observed, are shown in Tables 8–11, respectively. As seen in Tables 8–11, AEs other than those listed here may have had higher rates reported in either TPE or IVIG, but the differences were not significant, and sufficiently comparable studies could not be identified for a meta-analysis of >2 studies.

**Table 8:** Comparative vascular adverse event data for TPE in MG.

**References**	**Gajdos et al. (41)**	**Barth et al. (5)**	**Qureshi et al. (42)**	**Murthy et al. (47)**	**Köhler et al. (52)**	**Trikha et al. (65)**	**Rønager et al. (45)**	**Saeteng et al. (56)**	**El-Bawab et al. (64)**	**(1)**	**Guptill et al. (62)**	**Mandawat et al. (63)**	**Passero et al. (70)**
Treatment context	Acute	Acute	Acute	Acute	Acute	Acute	Chronic	Peri-op	Peri-op	Mixed	Mixed	Mixed	NS
									TPE		TPE		TPE
Comparison	TPE/	TPE/	TPE/	TPE/	TPE/	TPE QD/	TPE/	TPE/	all pts/	TPE/	Peripheral	TPE early/	citrate/
	IVIG	IVIG	IVIG	IVIG	lA	TPE QAD	IVIG	No TPE	TPE	MG	/TPE	TPE delayed	TPE
									selective		central^*^		heparin
Timing	15 d	30 d	hosp.	Tx	Tx	hosp.	16 wk	hosp.	hosp.	hosp.	Tx	hosp.	Tx
# Patients	41/46	41/40	28/26	15/8	10/9	16/17	12/12	33/53	74/90	1,269/340	100/34	870/183	72/107
Flow complication	-	10/0%	-	-	-	0/6%	-	-	5/2%	-	-	-	14/6%
Bleeding	-	-	-	-	-	0/0%	8/0%	3/0%	-	-	0/3%	-	-
Hemorrhage	-	-	-	-	0/11%	-	-	-	-	-	-	-	-
Hematoma	-	-	-	-	30/0%	-	-	-	-	-	0/6%	-	-
Coagulopathy	5/0%	-	4/0%	-	-	-	-	-	7/1%	-	-	-	-
Anemia	2/0%	0/3%	-	-	-	-	-	-	-	-	0/9%^∧^	-	-
Pancytopenia	-	-	-	-	10/0%	-	-	-	-	-	-	-	-
Deep Vein Thrombosis	-	-	-	-	-	13/6%	8/0%	-	-	-	0/12%^∧^	-	-
Pulmonary embolism	-	-	-	-	-	-	-	-	-	-	0/3%	-	-
Thrombosis (Unspecified)	2/0%	-	-	-	-	-	-	0/0%	-	2/1%	0/3%	1/1%	-
Syncope	-	5/0%	-	-	-	-	-	-	-	-	1/0%	-	-
Hypotension	5/0%	-	-	13/0%	20/11%	19/18%	17/0%	-	11/4%	-	-	-	-
Hypertension	-	0/3%	-	-	-	-	-	-	-	-	-	-	-
Vasospasm	-	20/0%^∧^	-	-	-	-	-	-	-	-	-	-	-

*hosp., during hospitalization; Tx, during treatment period; NS, not specified; lA, immunoadsorption; QD, daily; QAD, every other day. No significant difference observed between treatments (p ≥ 0.05) unless noted*.

∧ *p < 0.05 as reported by original authors or calculated using Fisher exact test;*

* *peripheral venous access and central venous access;^†^180 day follow up*.

**Table 9:** Comparative cardiac adverse event data for TPE in MG.

**References**	**Lal et al. (54)**	**Berrouschot et al. (53)**	**Gajdos et al. (41)**	**Barth et al. (5)**	**Qureshi et al. (42)**	**Köhler et al. (52)**	**Trikha et al. (65)**	**Mandawat et al. (1)**	**Guptill et al. (62)**	**Mandawat et al. (63)**	**Saeteng et al. (56)**
Treatment context	Acute	Acute	Acute	Acute	Acute	Acute	Acute	Mixed	Mixed	Mixed	Peri-op
Comparison	TPE/	TPE/	TPE/	TPE/	TPE /	TPE /	TPE QD/	TPE/	TPE peripheral/	TPE early	TPE/
	No TPE^**^	No TPE^**^	IVIG	IVIG	IVIG	lA	TPE Q.AD	IVIG	TPE central^*^	TPE delayed	No TPE
Timing	hosp.	NS	15 days	30 days	hosp.	Tx	hosp.	hosp.	Tx	hosp.	hosp.
# Patients	4/3	21/42	41/46	41/40	28/26	10/9	16/17	1,269/340	100/34	870/183	33/53
Cardiac complication (Unspecified)	-	-	-	-	21/4%	-	-	15/10%^∧^	-	12/25%^∧^	-
Arrhythmia (Other/Unspecified)	0/33%	29/12%	-	-	-	20/11%	-	-	1/15%^∧^	-	0/0%
Tachycardia	-	-	2/0%	-	-	10/33%	0/6%	-	-	-	-
Heart failure	-	-	-	2/0%	-	0/0%	-	-	-	-	-
Myocardial Infarction	-	-	-	2/0%	-	-	-	-	-	-	-

*hosp., during hospitalization; Tx, during treatment period; lA, immunoadsorption; QD, daily; Q.AD, every other day; NS, not stated. No significant difference observed between treatments (p ≥ 0.05) unless noted*.

∧ *p < 0.05 as reported by original authors or calculated using Fisher exact test*.

* *peripheral venous access and central venous access*.

** *Background treatment: (54) intravenous methyl prednisone, (53) pyridostigmine or pyridostigmine + prednisolone*.

**Table 10:** Comparative infection adverse event data for TPE in MG.

**Reference**	**Lal et al. (54)**	**Köhler et al. (53)**	**Barth et al. (5)**	**Pittayanon et al. (50)**	**Qureshi et al. (42)**	**Köhler et al. (52)**	**Schneider-Gold et al. (51)**	**Liew et al. (25)**	**Rønager et al. (45)**	**Sarkar et al. (55)**	**Saeteng et al. (56)**	**El-Bawab et al. (64)**	**Mandawat et al. (1)**	**Guptill et al. (62)**	**Mandawat et al. (63)**
Treatment context	Acute	Acute	Acute	Acute	Acute	Acute	Acute	Chronic	Chronic	Peri-op	Peri-op	Peri-op	Mixed	Mixed	Mixed
Comparison	TPE/No TPE^***^	TPE/No TPE^***^	TPE/IVIG	TPE/IVIG	TPE/IVIG	TPE/lA	TPE/lA	TPE/IVIG	TPE/IVIG	TPE/No TPE	TPE/No TPE	TPE all pts/TPEselective	TPE/IVIG	TPE peripheral/ TPE central^**^	TPE early/TPE delayed
Timing	hosp.	hosp.	30 d	hosp.	hosp.	180 d	hosp.	1 yr^*^	16 wk	hosp.	hosp.	hosp.	hosp.	Tx	hosp.
# Patients	4/3	21/42	41/40	21/9	28/26	10/9	19/24	17/20	12/12	10/9	33/53	74/90	1,269/340	100/34	870/183
Infection (unspecified/ other)	-	-	-	-	21/8%	0/11%	-	-	-	20/56%	0/2%	3/1%	5/1%^∧^	-	3/8%^∧^
Pneumonia	75/33%	24/14%	0/3%	19/11%	-	10/22%	5/8%	-	-	10/33%	-	-	-	-	-
Bacteremia	25/0%	-	-	-	-	-	-	-	-	-	-	-	-	1/3%	-
Sepsis	0/33%	5/0%	-	-	-	20/0%	-	6/0%	8/0%	-	-	-	-	1/3%	-

*hosp, during hospitalization; Tx, during treatment period; lA, immunoadsorption*.

*No significant difference observed between treatments (p ≥ 0.05) unless noted*.

∧ *p < 0.05 as reported by original authors or calculated using Fisher exact test*.

* *Timing: (44) through hospitalization or later, (25) median 1 yr follow-up (range 0-5 yrs), (10) mean 116 mo for all patients (100.2 ± 41.2 mo TPE vs. 125.1 ± 77.5 mo No TPE)*.

** *peripheral venous access and central venous access*.

*** *Background treatment: (54) intravenous methyl prednisone, (53) pyridostigmine or pyridostigmine + prednisolone*.

**Table 11:** Additional comparative adverse event data for TPE in MG.

**References**	**Gajdos et al. (41)**	**Barth et al. (5)**	**Qureshi et al. (42)**	**Murthyet al. (47)**	**Köhler et al. (52)**	**Trikha et al. (65)**	**Liew et al. (25)**	**Rønager et al. (45)**	**Saeteng et al. (56)**	**Mandawat et al. (1)**	**Citterio et al. (71)**	**Guptill et al. (62)**	**Mandawat et al. (63)**
Treatment context	Acute	Acute	Acute	Acute	Acute	Acute	Chronic	Chronic	Peri-op	Mixed	Mixed	Mixed	Mixed
												TPE	TPE
Comparison	TPE/	TPE/	TPE/	TPE/	TPE/	TPE QD/	TPE/	TPE/	TPE/	TPE/	TPE/	peripheral	early/
	IVIG	IVIG	IVIG	IVIG	lA	TPE QAD	IVIG	IVIG	No TPE	IVIG	IVIG	/TPE	TPE
												central^**^	delayed
Timing	15 days	30 days	hosp.	Tx	Tx	hosp.	1 yr^*^	16 wk	hosp.	hosp.	14 yrs^*^	Tx	hosp.
Patients	41/46	41/40	28/26	15/8	10/9	16/17	20/17	12/12	33/53	1,269/340	318/176	100/34	870/183
Renal Failure	-	-	-	-	-	-	-	-	-	5/1%^∧^	-	0/9%^∧^	1/4%^∧^
Hypokalemia	-	-	-	-	10/11%	-	-	-	-	-	-	-	-
Elevated BUN	-	-	0/8%	0/13%	-	-	-	-	-	-	-	-	-
Fever	5/0%	0/8%	-	-	30/0%	-	0/10%	0/33%	0/0%	-	-	-	-
Chills	5/0%	0/5%	-	-	-	-	-	-	-	-	-	-	-
Headache	0/2%	0/20%^∧^	-	-	-	-	-	0/58%^∧^	-	-	-	-	-
Nausea and vomiting	2/0%	0/18%^∧^	-	-	-	-	-	0/25%	-	-	-	-	-
Allergic reaction	-	0/5%	-	-	10/0%	6/0%	-	-	0/0%	-	-	-	-
Citrate reaction	-	15/0%^∧^	-	-	-	-	-	-	-	-	-	-	-
Extrathymic tumor	-	-	-	-	-	-	-	-	-	-	8/14%	-	-

*hosp., during hospitalization; Tx, during treatment period; IA, immunoadsorption. No significant difference observed between treatments (p ≥ 0.05) unless noted*.

∧ *p < 0.05 as reported by original authors or calculated using Fisher exact test*.

* *(25) median 1 yr follow-up (range 0–5 yrs), (71) 14 yr mean follow up across all patients*.

** *peripheral venous access and central venous access*.

##### PE vs. IVIG

AEs with significantly higher rates in TPE included cardiovascular AEs, infections, renal failure, and citrate reactions, while IVIG showed higher rates of extra-thymic tumor formation, headache, and nausea and vomiting.

In a combined analysis of all MG patients (crisis and non-crisis) in a retrospective HCUPNIS analysis (1), the adjusted odds ratio for any severe complication favored IVIG, but did not reach statistical significance (odds ratio IVIG/TPE: 0.71, *p* = 0.07). However, among myasthenic crisis patients treated with TPE or IVIG, the rates of cardiac complications, systemic infections, and acute renal failure were all significantly higher among TPE-treated patients. Cardiac complications, comprising hypotension, fluid overloading, arrhythmias, myocardial infarction, and cardiac arrest, was the most frequently observed category in both TPE and IVIG (22.68 vs. 11.83%, *p* = 0.001). The most significant difference between TPE and IVIG was observed in systemic infections, which included bacteremia, sepsis, systemic inflammatory response syndrome, and anaphylaxis (9.45 vs. 1.18%, *p* < 0.0001). Acute renal failure was significantly higher in the TPE cohort (4.73 vs. 1.18%, *p* = 0.038). In contrast, rates for non-crisis MG patients were lower for each category and no statistically significant differences were observed between TPE and IVIG (cardiac: 9.50 vs. 7.60%, *p* = 0.55; infection: 1.63 vs. 1.17%, *p* = 1.00; renal failure: 0.27 vs. 1.17%, *p* = 0.16; *N* = 737 and 171, respectively). A fourth AE category, thrombotic complications, exhibited a higher rate in TPE among crisis patients and a lower rate in TPE among non-crisis patients, though neither was statistically significant (crisis: 3.40 vs. 0.59%, *p* = 0.05; non-crisis: 0.27 vs. 0.58%, *p* = 0.46).

A prospective, randomized study comparing TPE (*N* = 41) and IVIG (*N* = 40) in acute MG found significant differences in the rate of several AEs (5, 72). Citrate reaction (14.6 vs. 0%, *p* = 0.03) and vasospasm (19.5 vs. 0%, *p* = 0.0054) were observed specifically in TPE. In contrast, headache (0 vs. 20.0%, *p* = 0.0024) and nausea & vomiting (0 vs. 17.5%, *p* = 0.0054) occurred solely in the IVIG group. A prospective controlled crossover comparison of TPE vs. IVIG as maintenance therapies similarly reported headache as an IVIG-specific adverse event (0 vs. 58%, *p* = 0.0046) (45).

A large, retrospective survey examined the risk factors associated with extrathymic tumor formation in 2,479 MG patients over long term follow-up (mean 14 years) (71). Increased risk of extrathymic tumor formation as a function of MG treatment history was summarized by odds ratios for several common MG treatments, including TPE (OR: 1.1, 95% CI 0.6–1.7) and IVIG (OR: 1.8, 95% CI 1.1–3.0). Among all treatments considered, only history of IVIG treatment was confirmed by multivariate logistic regression analysis to have a statistically significant association with extrathymic tumor formation (adjusted OR: 1.8, 95% CI 1.1–2.9).

##### Optimizing TPE

A few studies have also looked at the effects of procedural factors on specific adverse events, beyond mortality, in MG patients treated with TPE. Significantly better safety outcomes were achieved when TPE was performed soon after hospital admission and via peripheral venous access.

A retrospective study compared adverse event rates in TPE for MG performed via peripheral (*N* = 100) vs. central venous access (*N* = 34) (62). Cohorts included TPE use under any MG treatment context. Rates of several specific AEs were significantly lower for peripheral access: anemia or coagulopathy requiring transfusion (0 vs. 9%, *p* = 0.015), deep vein thrombosis (0 vs. 12%, *p* = 0.0033), arrhythmia (atrial fibrillation with rapid ventricular response: 1 vs. 15%, p = 0.0041), and acute renal failure (0 vs. 9%, *p* = 0.015).

The retrospective analysis of TPE timing within HCUPNIS found that the rates of several major AE categories were lower in early TPE (0–2 days from admission, *N* = 870) vs. delayed TPE (>2 days from admission, *N* = 183) (63). Specifically, the rates of cardiac complications (11.8 vs. 24.6%, *p* < 0.0001), systemic infections (2.9 vs. 7.7%, *p* < 0.001), and acute renal failure (1.0 vs. 3.8%, *p* = 0.009) were all statistically lower when TPE was performed early. The adjusted odds ratio for any complication from the above categories showed a significant increase in risk when TPE treatment was delayed (odds ratio delayed/early: 1.49, *p* < 0.0001).

##### COBE Spectra and Spectra Optia Apheresis Systems

As noted for efficacy and mortality, data comparing other adverse events associated with different TPE systems was not found in published literature. Tables 8–11 include adverse event data from several studies captured in this report that used the Spectra systems, COBE Spectra and Spectra Optia Apheresis Systems, exclusively (5, 62, 64, 70) or as one of two cited systems (51, 69). Safety findings from studies exclusively using COBE Spectra and Spectra Optia Apheresis Systems in comparison to IVIG (5, 72) and that used COBE Spectra Apheresis System exclusively in comparing between peripheral and central venous access (62) have been described in previous sections. Safety data in other TPE optimization studies using COBE Spectra Apheresis System reported higher rates of several AEs under certain conditions, but differences did not reach statistical significance in any case (64, 69, 70). Lastly, in a retrospective analysis comparing TPE vs. immunoadsorption in acute MG from an institution that utilized COBE Spectra Apheresis System as one of its two TPE systems, a higher overall adverse event rate was reported with TPE (36.9 vs. 4.2%, *p* < 0.05) (51). Other than pneumonia, for which there was no significant difference between the two treatments, rates of specific AEs were not reported.

## Limitations

There are several limitations of this report and the body of literature on which it is based. As has been noted by many authors in the past, establishing a baseline benefit of TPE is not possible from the available literature given the lack of placebo-controlled studies, likely due to the life-threatening nature of the condition in the acute context. Others have noted that decades of observational experience likely preclude and render unethical the performance of a randomized trial with a placebo arm (73, 74). As such, this report was designed to capture comparative data between TPE and other key treatments in use within MG. The included studies are mostly retrospective or prospective, but not blinded, not adjusted for potential geographic differences, and the majority do not specify the TPE system or separation method. Although we highlighted one system, comparison of other devices in the future would be beneficial. Other systematic reviews and some guidelines utilized more narrow inclusion criteria, which can be complemented by the more inclusive approach of this report. Another meta-analysis comparing plasmapheresis with IVIG looked strictly at prospective studies but also included other forms of plasmapheresis (75). Lastly, predominately retrospective or prospective studies were used in the analysis; randomized controlled studies would be preferred but unfortunately this is a limitation of available data.

Despite a comprehensive, inclusive approach, it is possible that our search may not have captured all relevant studies. Potential gaps include non-English articles, articles published prior to 1997, but not cited in later literature, articles not indexed in either of the databases utilized, or articles for which full text copies could not be obtained. Furthermore, the strategy was not designed to specifically look at data for MG patients seronegative for MuSK antibodies or positive for LRP 4, but this area remains of interest. Given the dearth of available data to determine if seropositive or seronegative patients should be treated differently with the available treatment, the currently available studies indicate that these patients should be treated similarly.

Regarding professional treatment guidelines, the most recent versions were given precedence. Several of these are more than 5 years old and more recent data might have altered the perspectives of their developers (4, 37).

In the meta-analyses, because of design limitations in the available studies, including variable disease scale, serotype, and timepoint, additional variables cannot be ruled out as potentially affecting TPE vs. IVIG outcomes. Baseline disease severity could also impact both efficacy and safety results, as was specifically noted in the largest retrospective analysis included in this report (1). It is also notable that in several instances an AE commonly mentioned in the literature to be associated with TPE was higher than a comparator, but the difference was not significant, which could be an unavoidable limitation of the small size of many of the included studies (Tables 8–11).

## Conclusions

TPE is an effective and appropriate MG treatment as evidenced by original research studies and professional guideline recommendations.

Although placebo controlled studies are not available, studies report high response rates among acute MG patients treated with TPE, as has been summarized previously (76). There is a large body of evidence associating TPE with improved disease severity and recovery from crisis in acute MG patients. In acute MG, a risk/benefit tradeoff appears to exist between TPE and IVIG. The meta-analysis results from this report indicate a higher response rate with TPE vs. IVIG in acute MG patients. The meta-analysis did not find differences in mortality; however, IVIG was associated with a lower risk of potentially serious adverse events. If peripheral rather than central venous access can be utilized in TPE treatment of myasthenic crisis, evidence shows a significant drop in most serious AE rates. Some evidence suggests that fast responses and shorter ventilation times are more likely with TPE, which might increase the favorability of TPE when a rapid response is particularly important. TPE might be preferred over IVIG in patients with a higher baseline risk of extrathymic tumor formation than the broader MG population, or in patients particularly sensitive to headache or nausea & vomiting. Conversely, IVIG might be preferred in patients with a higher baseline risk of cardiovascular AEs, infections, and renal failure than the broader MG population. It is important to note that these factors potentially impacting risk/benefit tradeoffs have not been evaluated prospectively but warrant further investigation. Irrespective of IVIG, peripheral venous access and early intervention are associated with significantly more favorable outcomes of TPE treatment in acute MG. There is direct of evidence that TPE is superior to IVIG in MuSK+ patients from a single comparative study, which is reflected in professional guidelines. Other studies in which TPE and IVIG are used in MuSK+ patients, but they do not provide clear comparative evidence and further investigation would be valuable.

Most published evidence has demonstrated that outcomes in patients undergoing thymectomy are superior for those who receive TPE prior to surgery compared to those who do not receive acute immunomodulatory therapy. However, there is no available evidence to suggest that TPE has superior efficacy over IVIG when used pre-thymectomy, and TPE vs. IVIG adverse event rates have not been reported in this context.

In maintenance therapy for chronic MG, the overall body of evidence comparing TPE to other treatments is quite limited and the limitation of the transient response is burdensome. In juvenile patients, there is evidence supporting the use of TPE over IVIG for maintenance therapy, but further investigation is needed beyond the single published comparative study. However, the relatively high response rates and lack of a statistically significant safety downsides to IVIG in available maintenance studies suggest that this treatment area warrants further investigation to further establish both comparative efficacy and safety.

In summary, available literature and professional recommendations strongly indicate that TPE has clear clinical utility in the treatment of acute MG and in improving thymectomy outcomes. There are notable side effects that should be considered, but neither a significantly increased risk of mortality nor a consistent pattern of serious adverse events exists across studies. TPE outcomes can be improved further with early intervention and peripheral venous access.

## Data Availability Statement

The raw data supporting the conclusions of this article will be made available by the authors, without undue reservation.

## Author Contributions

TSI and JSR: study concept, design, and drafting of the manuscript. All authors contributed to critically revising the manuscript.

## Conflict of Interest

TSI and JSR are consultants for Terumo Blood and Cell Technologies. ARD was an employee of Terumo Blood and Cell Technologies.

## Publisher's Note

All claims expressed in this article are solely those of the authors and do not necessarily represent those of their affiliated organizations, or those of the publisher, the editors and the reviewers. Any product that may be evaluated in this article, or claim that may be made by its manufacturer, is not guaranteed or endorsed by the publisher.

## Footnotes

^1^9 of the 165 articles were added from citations during full-text review.

^2^AAN scale “Level U” recommendation – very low confidence in evidence.

^3^One study reported a higher rate for TPE vs. IVIG when response was defined as clinically meaningful improvement in the primary outcome metric (ΔQMGS≥3.5) (58 vs. 51%), but an opposing trend when response was defined as an MGFA-PIS of improved or better (65 vs. 69%).

^4^Direct statistical comparison between TPE and IVIG not reported and not possible from published data because no variance metric was reported.

^5^The study did not report on the statistical differences between the efficacies of two treatments as measured by AChR titers.

^6^ΔQMGS ≥ 3.5: 57% response rate, MGFA-PIS “improved” or better: 65% response rate.

^7^HCUPNIS analysis comprised a mixed cohort (“Myasthenia gravis”), which was excluded from the meta-analysis, and an acute cohort (“myasthenia gravis crisis”), which was included in the meta-analysis.

^8^Includes any other specific AEs meeting at least one of the following criteria (1) appearing in >1 publication, (2) associated with TPE according to other existing literature, (3) subjectively evaluated by report authors as potentially serious (e.g., vasospasm, extrathymic tumors).

## References

[1] Mandawat A Kaminski HJ Cutter G Katirji B Alshekhlee A. Comparative analysis of therapeutic options used for myasthenia gravis. Ann. Neurol. (2010) 68:797–805. 10.1002/ana.22139 21061395

[2] Sanders DB Wolfe GI Benatar M Evoli A Gilhus NE Illa I. International consensus guidance for management of myasthenia gravis: Executive summary. Neurology. (2016) 87:419–25. 10.1212/WNL.0000000000002790 27358333 PMC4977114

[3] Abbas Jowkar C Lorenzo N. Myasthenia gravis: practice essentials, background, anatomy. Medscape. (2017). Available online at: https://emedicine.medscape.com/article/1171206-overview (accessed September, 2019).

[4] Skeie GO Apostolski S Evoli A Gilhus NE Illa I Harms L. Guidelines for treatment of autoimmune neuromuscular transmission disorders. Eur J Neurol. (2010) 17:893–902. 10.1111/j.1468-1331.2010.03019.x 20402760

[5] Barth D Nabavi Nouri M Ng E Nwe P Bril V. Comparison of IVIg and PLEX in patients with myasthenia gravis. Neurology. (2011) 76:2017–23. 10.1212/WNL.0b013e31821e5505 21562253 PMC3109880

[6] Zisimopoulou P Evangelakou P Tzartos J Lazaridis K Zouvelou V Mantegazza R. A comprehensive analysis of the epidemiology and clinical characteristics of anti-LRP4 in myasthenia gravis. J Autoimmun. (2014) 52:139–45. 10.1016/j.jaut.2013.12.004 24373505

[7] NIH. Myasthenia Gravis Fact Sheet. (2017). Available online at: https://www.ninds.nih.gov/Disorders/Patient-Caregiver-Education/Fact-Sheets/Myasthenia-Gravis-Fact-Sheet (accessed September, 2019).

[8] Linda M Wendell C Joshua Levine M. Myasthenic crisis neuroanesthesia neurocritical care. Case Stud. (2011) 1:321–3. 10.1177/1941875210382918 23983833 PMC3726100

[9] Alipour-Faz A Shojaei M Peyvandi H Ramzi D Oroei M Ghadiri F. A comparison between IVIG and plasma exchange as preparations before thymectomy in myasthenia gravis patients. Acta Neurol Belg. (2017) 117:245–9. 10.1007/s13760-016-0689-z 27530310

[10] Nagayasu T Yamayoshi T Matsumoto K Ide N Hashizume S Nomura M. Beneficial effects of plasmapheresis before thymectomy on the outcome in myasthenia gravis. Jpn J Thorac Cardiovasc Surg. (2005) 53:2–7. 10.1007/s11748-005-1001-y 15724495

[11] Guptill JT Juel VC Massey JM Anderson AC Chopra M Yi JS. Effect of therapeutic plasma exchange on immunoglobulins in myasthenia gravis. Autoimmunity. (2016) 49:472–9. 10.1080/08916934.2016.1214823 27684107 PMC5440840

[12] Reeves HM Winters JL. The mechanisms of action of plasma exchange. Br J Haematol. (2014) 164:342–51. 10.1111/bjh.12629 24172059

[13] Lehmann HC Hartung HP. Plasma exchange and intravenous immunoglobulins: Mechanism of action in immune-mediated neuropathies. J Neuroimmunol. (2011) 231:61–9. 10.1016/j.jneuroim.2010.09.015 21056913

[14] Dhawan PS Goodman BP Harper CM Bosch PE Hoffman-Snyder CR Wellik KE. IVIG versus PLEX in the treatment of worsening myasthenia gravis: What is the evidence? A critically appraised topic. Neurologist. (2015) 19:145–8. 10.1097/NRL.0000000000000026 25970838

[15] Gajdos P Chevret S Toyka K. Intravenous immunoglobulin for myasthenia gravis. Cochrane Database Syst Rev. (2003) 12:CD002277. 10.1002/14651858.CD002277 12804431

[16] McDaneld LM Fields JD Bourdette DN Bhardwaj A. Immunomodulatory therapies in neurologic critical care. Neurocrit Care. (2010) 12:132–43. 10.1007/s12028-009-9274-0 19774497

[17] Gajdos P Chevret S Toyka K. Intravenous immunoglobulin for myasthenia gravis. Cochrane Database Syst Rev. (2006) 12:CD002277. 10.1002/14651858.CD002277.pub2 16625559

[18] Dalakas MC. Intravenous Immunoglobulin in autoimmune neuromuscular diseases. JAMA. (2004) 291:2367–75. 10.1001/jama.291.19.2367 15150209

[19] Winters JL Pineda AA. New directions in plasma exchange. Curr Opin Hematol. (2003) 10:424–8. 10.1097/00062752-200311000-00005 14564172

[20] Gajdos P Chevret S Toyka K. Plasma exchange for generalised myasthenia gravis. Cochrane Database Syst Rev. (2002) 4:2002. 10.1002/14651858.CD002275 12519572 PMC8985203

[21] Gajdos P Chevret S Toyka KV. Intravenous immunoglobulin for myasthenia gravis. Cochrane Database Syst Rev. (2012) 12:CD002277. 10.1002/14651858.CD002277.pub4 23235588 PMC7133495

[22] Gajdos P Chevret S. Treatment of myasthenia gravis acute exacerbations with intravenous immunoglobulin. Ann N Y Acad Sci. (2008) 1132:271–5. 10.1196/annals.1405.001 18096850

[23] Gajdos P Chevret S Toyka KV. Intravenous immunoglobulin for myasthenia gravis. Cochrane Database Syst Rev. (2008) 1:CD002277. 10.1002/14651858.CD002277.pub3 18254004

[24] Ng JK Ng CS Underwood MJ Lau KK. Does repeat thymectomy improve symptoms in patients with refractory myasthenia gravis?. Interact Cardiovasc Thorac Surg. (2014) 8:376–80. 10.1093/icvts/ivt493 24532639 PMC3930209

[25] Liew WK Powell CA Sloan SR Shamberger RC Weldon CB Darras BT. Comparison of plasmapheresis and intravenous immunoglobulin as maintenance therapies for juvenile myasthenia gravis. JAMA Neurol. (2014) 71:575–80. 10.1001/jamaneurol.2014.17 24590389

[26] Shamseer L Moher D Clarke M Ghersi D Liberati A Petticrew M. PRISMA-P (preferred reporting items for systematic review and meta-analysis protocols) 2015 checklist : recommended items to address in a systematic review protocol. BMJ. (2015) 349:2015. 10.1136/bmj.g7647 25555855

[27] Iani C Caramia M Morosetti M Loberti M Palmieri MG Meloni C. The treatment of severe forms of myasthenia gravis. Funct Neurol. (1998) 13:231–7. 9800150

[28] Tireli H Karlikaya G Tutkavul K Akpinar A Okay T. Myasthenia gravis: how to treat?. Myopathies cardiomyopathies Off. J Mediterr Soc Myol. (2004) 23:140–5. 15938570

[29] Pérez Nellar J Domínguez AM Llorens-Figueroa JA Ferrá-Betancourt A Pardo A Quiala M. [A comparative study of intravenous immunoglobulin and plasmapheresis preoperatively in myasthenia]. Rev Neurol. (2001) 33:413–6. 10.33588/rn.3305.2001132 11727205

[30] DerSimonian R Laird N. Meta-analysis in clinical trials revisited. Contemp Clin Trials. (2015) 45:139–45. 10.1016/j.cct.2015.09.002 26343745 PMC4639420

[31] Egger M Davey Smith G Schneider M Minder C. Bias in meta-analysis detected by a simple, graphical test. BMJ. (1997) 315:629–34. 10.1136/bmj.315.7109.629 9310563 PMC2127453

[32] Skeie GO Apostolski S Evoli A Gilhus NE Illa I Harms L. Guidelines for the treatment of autoimmune neuromuscular transmission disorders. Eur J Neurol. (2006) 13:691–9. 10.1111/j.1468-1331.2006.01476.x 16834699

[33] Padmanabhan A Connelly-Smith L Aqui N Balogun RA Klingel R Meyer E. Guidelines on the use of therapeutic apheresis in clinical practice-evidence-based approach from the writing committee of the American society for apheresis: the seventh special issue. J Clin Apheresis. (2016) 31:149–62. 10.1002/jca.21470 27322218

[34] Schwartz J Winters JL Padmanabhan A Balogun RA Delaney M Linenberger ML. Guidelines on the use of therapeutic apheresis in clinical practice-evidence-based approach from the writing committee of the American society for apheresis: the sixth special issue. J Clin Apher. (2013) 28:145–284. 10.1002/jca.21276 23868759

[35] Szczepiorkowski ZM Winters JL Bandarenko N Kim HC Linenberger ML Marques MB. Guidelines on the use of therapeutic apheresis in clinical practice - Evidence-based approach from the apheresis applications committee of the American Society for Apheresis. J Clin Apheresis. (2010) 22:106–75. 17394188 10.1002/jca.20129

[36] Szczepiorkowski ZM Bandarenko N Kim HC Linenberger ML Marques MB Sarode R. Guidelines on the use of therapeutic apheresis in clinical practice: evidence-based approach from the Apheresis Applications Committee of the American Society for Apheresis. J Clin Apher. (2007) 22:106–75. 10.1002/jca.20129 17394188

[37] Cortese I Chaudhry V So YT Cantor F Cornblath DR Rae-Grant A. Evidence-based guideline update : plasmapheresis in neurologic disorders report of the therapeutics and technology assessment. Neurology. (2011) 76:294–300. 10.1212/WNL.0b013e318207b1f6 21242498 PMC3034395

[38] The utility of therapeutic plasmapheresis for neurological disorders. NIH Consensus Development. JAMA. (1986) 256:1333–7. 10.1001/jama.256.10.1333 3747048

[39] Diener HC Weimar C Berlit P Deuschl G Elger C Gold R. Leitlinien für Diagnostik und Therapie in der Neurologie. Stuttgart: Georg Thieme Verlag (2008).

[40] Klingel R Heibges A Fassbender C. Plasma exchange and immunoadsorption for autoimmune neurologic diseases - current guidelines and future perspectives. Atheroscler. (2009) 10:129–32. 10.1016/S1567-5688(09)71827-6 20129391

[41] Gajdos P Chevret S Clair B Tranchant C Chastang C. Clinical trial of plasma exchange and high-dose intravenous immunoglobulin in myasthenia gravis. Myasthenia Gravis Clinical Study Group. Ann Neurol. (1997) 41:789–96. 10.1002/ana.410410615 9189040

[42] Qureshi AI Choudhry MA Akbar MS Mohammad Y Chua HC Yahia AM. Plasma exchange versus intravenous immunoglobulin treatment in myasthenic crisis. Neurology. (1999) 52:629. 10.1212/WNL.52.3.629 10025801

[43] Ramos-Fransi A Rojas-García R Segovia S Márquez-Infante C Pardo J Coll-Cantí J. Myasthenia gravis: Descriptive analysis of life-threatening events in a recent nationwide registry. Eur J Neurol. (2015) 22:1056–61. 10.1111/ene.12703 25847221

[44] Guptill JTT Sanders DBB Evoli A. Anti-MuSK antibody myasthenia gravis: clinical findings and response to treatment in two large cohorts. Muscle Nerve. (2011) 44:36–40. 10.1002/mus.22006 21674519

[45] Rønager J Ravnborg M Hermansen I Vorstrup S. Immunoglobulin treatment versus plasma exchange in patients with chronic moderate to severe myasthenia gravis. Artif Organs. (2001) 25:967–731. 10.1046/j.1525-1594.2001.06717.x 11843764

[46] Katzberg HDD Barnett C Bril V. Predictors of response to immunomodulation in patients with myasthenia gravis. Muscle Nerve. (2012) 45:648–52. 10.1002/mus.23236 22499090

[47] Murthy J Meena A Chowdary G Naryanan J. Myasthenic crisis: clinical features, complications and mortality. Neurol India. (2005) 53:37. 10.4103/0028-3886.15050 15805653

[48] Barnett C Wilson G Barth D Katzberg HD Bril V. Changes in quality of life scores with intravenous immunoglobulin or plasmapheresis in patients with myasthenia gravis. J Neurol Neurosurg Psychiatry. (2013) 84:94–7. 10.1136/jnnp-2011-301449 23154126

[49] Liu Z Yao S Zhou Q Deng Z Zou J Feng H. Predictors of extubation outcomes following myasthenic crisis. J Int Med Res. (2016) 44:1524–33. 10.1177/0300060516669893 27856933 PMC5536745

[50] Pittayanon R Treeprasertsuk S Phanthumchinda K. Plasmapheresis or intravenous immunoglobulin for myasthenia gravis crisis in King Chulalongkorn Memorial Hospital. J Med Assoc Thail. (2009) 92:478–82. 19374297

[51] Schneider-Gold C Krenzer M Klinker E Mansouri-Thalegani B Müllges W Toyka KV. Immunoadsorption versus plasma exchange versus combination for treatment of myasthenic deterioration. Ther Adv Neurol Disord. (2016) 9:297–303. 10.1177/1756285616637046 27366236 PMC4916519

[52] Köhler W Bucka C Klingel R. A randomized and controlled study comparing immunoadsorption and plasma exchange in myasthenic crisis. J Clin Apher. (2011) 26:347–55. 10.1002/jca.20317 22095647

[53] Berrouschot J Baumann I Kalischewski P Sterker M Schneider D. Therapy of myasthenic crisis. Crit Care Med. (1997) 25:1228–35. 10.1097/00003246-199707000-00027 9233752

[54] Lal V Prabhakar S Agarwal R Sharma S. Clinical profile and outcome of myasthenic crisis in a tertiary care hospital: A prospective study. Ann Indian Acad Neurol. (2013) 16:203. 10.4103/0972-2327.112466 23956564 PMC3724074

[55] Sarkar BKK Sengupta P Sarkar UNN. Surgical outcome in thymic tumors with myasthenia gravis after plasmapheresis - a comparative study. Interact Cardiovasc Thorac Surg. (2008) 7:1007–10. 10.1510/icvts.2007.170894 18682429

[56] Saeteng S Tantraworasin A Siwachat S Lertprasertsuke N Euathrongchit J Wannasopha Y. Preoperative plasmapheresis for elective thymectomy in myasthenia patient: is it necessary?. ISRN Neurol. (2013) 2013:238783. 10.1155/2013/238783 23840964 PMC3693175

[57] d'Empaire G Hoaglin DC Perlo VP Pontoppidan H. Effect of prethymectomy plasma exchange on postoperative respiratory function in myasthenia gravis. J Thorac Cardiovasc Surg. (1985) 89:592–6. 10.1016/S0022-5223(19)38763-X 3982061

[58] Jensen P Bril V. Short report a comparison of the effectiveness of intravenous immunoglobulin and plasma exchange as preoperative therapy of myasthenia gravis. J Clin Neuromuscul Dis. (2008) 9:352–5. 10.1097/CND.0b013e3181660807 18344718

[59] Goti P Spinelli A Marconi G Duranti R Gigliotti F Pizzi A. Comparative effects of plasma exchange and pyridostigmine on respiratory muscle strength and breathing pattern in patients with myasthenia gravis. Thorax. (1995) 50:1080–6. 10.1136/thx.50.10.1080 7491557 PMC475022

[60] Ohta K Shigemoto K Fujinami A Maruyama N Konishi T Ohta M. Clinical and experimental features of MuSK antibody positive MG in Japan. Eur J Neurol. (2007) 14:1029–34. 10.1111/j.1468-1331.2007.01870.x 17718696

[61] Gajdos P Simon N de Rohan-Chabot P Raphael JC Goulon M. Long-term effects of plasma exchange in myasthenia. Results of a randomized study. Presse Med. (1983) 12:939–42. 6221247

[62] Guptill JT Oakley D Kuchibhatla M Guidon AC Hobson-Webb LD Massey JM. A Retrospective study of complications of therapeutic plasma exchange in myasthenia. Muscle Nerve. (2013) 47:170–6. 10.1002/mus.23508 23168720

[63] Mandawat AAA Mandawat AAA Kaminski HJJ Shaker ZAA Alawi AAA Alshekhlee A. Outcome of plasmapheresis in myasthenia gravis: Delayed therapy is not favorable. Muscle Nerve. (2011) 43:578–84. 10.1002/mus.21924 21404289

[64] El-Bawab H Hajjar W Rafay M Bamousa A Khalil A Al-Kattan K. Plasmapheresis before thymectomy in myasthenia gravis: routine versus selective protocols. Eur J Cardio-Thoracic Surg. (2009) 35:392–7. 10.1016/j.ejcts.2008.11.006 19136275

[65] Trikha I Singh S Goyal V Shukla G Bhasin R Behari M. Comparative efficacy of low dose, daily versus alternate day plasma exchange in severe myasthenia gravis: A randomised trial. J Neurol. (2007) 254:989–95. 10.1007/s00415-006-0235-7 17694386

[66] Hawkey CJ Newsom-davis J Vincent A. Plasma exchange and immunosuppressive drug treatment in myasthenia gravis : no evidence for synergy. J Neurol Neurosurg Psychiatry. (1981) 1981:469–75. 10.1136/jnnp.44.6.469 7276959 PMC491026

[67] Pasnoor M Wolfe GI Nations S Trivedi J Barohn RJ Herbelin L. Clinical findings in MuSK-antibody positive myasthenia gravis: a U.S. experience. Muscle Nerve. (2010) 41:370–4. 10.1002/mus.21533 19882635

[68] Yamada C Pham HP Wu Y Cooling L Kim HC Morgan S. Report of the ASFA apheresis registry on muscle specific kinase antibody positive myasthenia gravis. J Clin Apher. (2017) 32:5–11. 10.1002/jca.21454 26946363

[69] Rock G Sutton DM Freedman J Nair RC. Pentastarch instead of albumin as replacement fluid for therapeutic plasma exchange. J Clin Apher. (1997) 12:165–9. 10.1002/(SICI)1098-1101(1997)12:4<165::AID-JCA2>3.0.CO;2-8 9483177

[70] Passero BA Zappone P Lee HE Novak C Maceira EL Naber M. Citrate versus heparin for apheresis catheter locks: An efficacy analysis. J Clin Apher. (2015) 30:22–7. 10.1002/jca.21346 25132635

[71] Citterio A Beghi E Millul A Evoli A Mantegazza R Antozzi C. Risk factors for tumor occurrence in patients with myasthenia gravis. J Neurol. (2009) 256:1221–7. 10.1007/s00415-009-5091-9 19330280

[72] Ebadi H Barth D Bril V. Safety of plasma exchange therapy in patients with myasthenia gravis. Muscle Nerve. (2013) 47:510–4. 10.1002/mus.23626 23322564

[73] Chhibber V Weinstein R. Evidence-based review of therapeutic plasma exchange in neurological disorders. Semin Dial. (2012) 25:132–9. 10.1111/j.1525-139X.2011.01023.x 22277020

[74] Kaminski HJ Cutter G Ruff R. Practice parameters and focusing research: plasma exchange for myasthenia gravis. Muscle Nerve. (2011) 43:625–6. 10.1002/mus.22080 21484820

[75] Ortiz Salas PA Gaviria Carrillo M Cortés Bernal GA Moreno Medina K Roa LF Rodríguez Quintana JH. Human immunoglobulin versus plasmapheresis in guillain-barre syndrome and myasthenia gravis. J Clin Neuromuscul Dis. (2016) 18:1–11. 10.1097/CND.0000000000000119 27552383

[76] Kuks JBM Skallebaek D. Plasmapheresis in myasthenia gravis. A survey. Transfus Sci. (1998) 19:129–36. 10.1016/S0955-3886(98)00022-8 10187038

